# Systematic review with meta-analysis of the epidemiological evidence relating FEV_1_ decline to lung cancer risk

**DOI:** 10.1186/1471-2407-12-498

**Published:** 2012-10-27

**Authors:** John S Fry, Jan S Hamling, Peter N Lee

**Affiliations:** 1 P N Lee Statistics and Computing Ltd, Sutton, Surrey, United Kingdom

## Abstract

**Background:**

Reduced FEV_1_ is known to predict increased lung cancer risk, but previous reviews are limited. To quantify this relationship more precisely, and study heterogeneity, we derived estimates of β for the relationship RR(diff) = exp(βdiff), where diff is the reduction in FEV_1_ expressed as a percentage of predicted (FEV_1_%P) and RR(diff) the associated relative risk. We used results reported directly as β, and as grouped levels of RR in terms of FEV_1_%P and of associated measures (e.g. FEV_1_/FVC).

**Methods:**

Papers describing cohort studies involving at least three years follow-up which recorded FEV_1_ at baseline and presented results relating lung cancer to FEV_1_ or associated measures were sought from Medline and other sources. Data were recorded on study design and quality and, for each data block identified, on details of the results, including population characteristics, adjustment factors, lung function measure, and analysis type. Regression estimates were converted to β estimates where appropriate. For results reported by grouped levels, we used the NHANES III dataset to estimate mean FEV_1_%P values for each level, regardless of the measure used, then derived β using regression analysis which accounted for non-independence of the RR estimates. Goodness-of-fit was tested by comparing observed and predicted lung cancer cases for each level. Inverse-variance weighted meta-analysis allowed derivation of overall β estimates and testing for heterogeneity by factors including sex, age, location, timing, duration, study quality, smoking adjustment, measure of FEV_1_ reported, and inverse-variance weight of β.

**Results:**

Thirty-three publications satisfying the inclusion/exclusion criteria were identified, seven being rejected as not allowing estimation of β. The remaining 26 described 22 distinct studies, from which 32 independent β estimates were derived. Goodness-of-fit was satisfactory, and exp(β), the RR increase per one unit FEV_1_%P decrease, was estimated as 1.019 (95%CI 1.016-1.021). The estimates were quite consistent (I^2^ =29.6%). Mean age was the only independent source of heterogeneity, exp(β) being higher for age <50 years (1.024, 1.020-1.028).

**Conclusions:**

Although the source papers present results in various ways, complicating meta-analysis, they are very consistent. A decrease in FEV_1_%P of 10% is associated with a 20% (95%CI 17%-23%) increase in lung cancer risk.

## Background

There have been a number of studies that have reported a strong relationship of forced expiratory volume in one second (FEV_1_) to risk of lung cancer (e.g. [1-10]). However, apart from a review in 2005 by Wasswa-Kintu *et al.*[11] we are unaware of any previous attempt to meta-analyse the available data, and that review restricted its meta-analysis only to those four studies which reported results by quintiles of FEV_1_, although noting the existence of data from a larger number of studies. In order to obtain a more precise estimate of the relationship of FEV_1_ to lung cancer risk, and to study factors which might affect the strength of this relationship, this systematic review and meta-analysis combines separate quantitative estimates of the relationship from studies which have presented their findings in a variety of ways. For each available set of data we estimate the slope (β) and its standard error (SE β) of the relationship RR(diff) = exp(βdiff) where diff is the reduction in FEV_1_ expressed as a percentage of its predicted value (FEV_1_%P), and RR(diff) is the relative risk associated with this reduction. Our procedures allow us to incorporate results reported as quintiles, by other grouped levels or as regression coefficients and also to include results reported not only in terms of FEV_1_%P, but also in terms of associated measures such as FEV_1_, or the ratio of FEV_1_ to forced vital capacity (FEV_1_/FVC).

## Methods

### Inclusion and exclusion criteria

Attention was restricted to epidemiological studies of cohort design involving a follow-up period of at least three years, in which FEV_1_ was recorded at baseline, and which presented the results of analyses relating FEV_1_ (or related measures) to subsequent risk of lung cancer.

The following exclusion criteria were applied:

#### Patients

Studies of patients who had undergone, or were selected for, surgery; of patients with cancer or serious diseases other than COPD; publications describing case reports or reviews concerning treatment for cancer or surgical procedures.

### Not cohort

Clinical studies; studies of cross-sectional design; studies involving a follow-up period shorter than three years.

#### Not lung cancer

Lung cancer not an endpoint; no lung cancer cases seen during follow-up.

#### Reviews not of interest

Review papers where the relationship of FEV_1_ to lung cancer was not considered, the papers typically only describing the relationship of an exposure (e.g. smoking) with FEV_1_ and separately with lung cancer.

Note that the four sets of exclusion criteria were applied in turn, and once one criterion was satisfied no attempt was made to consider the others.

### Literature searching

A Medline search was first carried out using the search term (“Forced expiratory volume” [Mesh Terms] OR FEV1 [All fields] OR “Forced expiratory volume” [All Fields]) AND Lung cancer) with no limits. An Embase search was then carried out using the same search terms. Reviews of interest, including the earlier systematic review of Wasswa-Kintu *et al.*[11], were then examined to see if they cited additional relevant references. Finally, reference lists of the papers obtained were examined.

### Identification of studies

Relevant papers were allocated to studies, noting multiple papers on the same study, and papers reporting on multiple studies. Each study was given a unique reference code (REF) of up to six characters (e.g. MANNIN or MRFIT), usually based on the principal author’s name. Possible overlaps between study populations were considered.

### Data recorded

Relevant information was entered onto a study database and a linked relative risk (RR) database. The study database contained a record for each study describing the following aspects: relevant publications; study title; study design; sexes considered; age range; details of the population studied; location; timing; length of follow-up; definition of lung cancer, and whether mortality or incidence. It also contains details of the individual components making up the Newcastle-Ottawa study quality score [12], described in detail in Additional file 1: Quality.

The RR database holds the detailed results, typically containing multiple records for each study. Each record is linked to the relevant study and refers to a specific RR, recording the comparison made and the results. This record includes the following: sex; age range; race; smoking status; adjustment factors; type of lung cancer; source publication and length of follow-up. For studies which provided a block of results by level of FEV_1_%P (or by an associated measure, such as FEV_1_/FVC, FEV_1_ unnormalised or SDs of FEV_1_/height^3^ below average), the record also included the measure reported, the range (or mean if provided) of values for the comparison group, and for each level the range (or mean) of values, and the reported or estimated RR and 95% confidence interval (CI) relative to the comparison group. Also recorded was an estimate of the ratio of the number at risk in the comparison group to the overall number at risk, and the ratio of the number at risk to the number of lung cancer cases for the block, and information to distinguish between multiple blocks within the same study (e.g. for different sexes or smoking groups). For studies which only provided summary statistics for a block (such as the RR for a 1% decrease in the measure), the record contained details of the summary statistic and also the information to distinguish between multiple blocks. Although our main analyses are restricted to the most relevant estimates recorded in the RR database (e.g. data for FEV_1_%P if available, direct estimates of β rather than estimates derived from RRs by level, data for longest follow-up, or whole population data rather than data for small subsets of the population), all data were entered as available. However, most studies did not allow any choice.

### Statistical methods

#### The basic model

The underlying model is that proposed by Berlin *et al.*[13], which we previously used to study the relationship of dose of environmental tobacco smoke exposure to lung cancer [14]. In this model, the absolute risk of lung cancer, R, in someone exposed to a given dose is expressed as

$$R=\alpha \exp\left(\beta d\right)$$

where α and β are constants. This implies that the relative risk RR(d_2_,d_1_) comparing dose d_2_ to dose d_1_ is given by

$$\begin{array}{l} \text{RR}\left({\text{d}}_{2},{\text{d}}_{1}\right)=\text{exp}\left(\beta \left({\text{d}}_{2}-{\text{d}}_{1}\right)\right)\\ \text{or RR}\left(\text{diff}\right)=\text{exp}\;(\beta \text{diff}) \end{array}$$

where diff is the difference in dose. This model implies that a fixed difference in dose increases risk by a fixed multiplicative factor.

When applying this model the dose, d, is the estimated mean level of FEV_1_%P, and the difference in doses, diff, is taken to be the reduction in FEV_1_%P compared to the highest level studied. As RRs tend to increase with decreasing level of FEV_1_%P, expressing diff in terms of reductions in FEV_1_%P ensures that estimates of β tend to be positive. Note that no attempt is made to estimate absolute risks or the parameter α, only the slope parameter, β, being estimated.

To use this method it was required to estimate β, and its standard error (SE β), for each block to be analysed. Three main situations were found in the blocks examined:

a) Some studies actually presented estimates of β together with its SE or 95% CI that could be used directly. Others presented estimates in a form that could readily be converted, e.g. increase in risk per 1% decrease in FEV_1_%P.

b) Other studies presented data by grouped values of FEV_1_%P either directly as RRs and 95% CIs or in other ways that allowed RRs and 95% CIs to be calculated using standard methods [15]. Berlin *et al.*[13] described a method for estimating β, and its standard error (SE β), that requires data for a study to consist of dose and number of cases and controls (or subjects at risk) at each level of exposure. The method is not a straightforward regression, as it has to take into account the fact that the level-specific RR estimates for a block are correlated, as they all depend on the same comparison group. It can also be applied to studies with data in the form of confounder-corrected RRs and 95% CIs, provided that such data are first converted into counts (“pseudo-numbers”). We used the method of Hamling *et al.*[16] to estimate the pseudo-numbers.

c) A final group of studies had RRs that were not expressed in terms of FEV_1_%P, but in terms of an associated measure, such as uncorrected FEV or FEV_1_/FVC. To ensure consistency in the estimation process for β, we converted values of the associated measure into values in terms of FEV_1_%P. To do this we made use of the publicly available data in the NHANES III study.

#### The NHANES III dataset

The National Health and Nutrition Examination Surveys (NHANES) were conducted on nationwide probability samples of approximately 32,000 persons 1–74 years of age. The NHANES III survey [17], conducted from 1988 to 1994, was the seventh in a series of these surveys based on a complex, multi-stage plan, designed to provide national estimates for the US of the health and nutritional status of the civilian, non-institutionalised population aged two months and older. *Inter alia*, the NHANES III study makes available data on age, sex, race, height, smoking habits, FEV_1_ and FVC on an individual-person basis.

Based on the NHANES data, Hankinson *et al.* (1999) [18] provides widely-used equations to predict FEV_1_ for an individual which are of the form:

$$\begin{array}{ll} {\text{FEV}}_{1}\left(\text{predicted}\right)= & {\text{b}}_{0}+{\text{b}}_{1}\text{age}\left(\text{years}\right)+{\text{b}}_{2}\text{age}{\left(\text{years}\right)}^{2}\\ & +{\text{b}}_{3}\text{height}{\left(\text{cm}\right)}^{2} \end{array}$$

where the coefficients: b_0_, b_1_, and b_2,_ vary by sex, race and age, as shown in Table 1. The observed value of FEV_1_ for an individual can then be divided by the predicted value based on the individual’s characteristics, and then multiplied by 100, to give the estimated value of FEV_1_%P for that individual. 

**Table 1:** **Age, sex and race specific coefficients used to predict FEV_1_ for the equations of Hankinson *et al. ***[18]^**a**^

**Sex**	**Race**	**Age**	**b**_**0**_	**b**_**1**_	**b**_**2**_	**b**_**3**_
Male	Caucasian	<20	−0.7453	−0.04106	0.004477	0.00014098
		20+	0.5536	−0.01303	−0.000172	0.00014098
	African-American	<20	−0.7048	−0.05711	0.004316	0.00013194
		20+	0.3411	−0.02309	0	0.00013194
	Mexican-American	<20	−0.8218	−0.04248	0.004291	0.00015104
		20+	0.6306	−0.02928	0	0.00015104
Female	Caucasian	<18	−0.8710	0.06537	0	0.00011496
		18+	0.4333	−0.00361	−0.000194	0.00011496
	African-American	<18	−0.9630	0.05799	0	0.00010846
		18+	0.3433	−0.01283	−0.000097	0.00010846
	Mexican-American	<18	−0.9641	0.06490	0	0.00012154
		18+	0.4529	−0.01178	−0.000113	0.00012154

a The equation is of the form: FEV_1_ (predicted) = b0 + b1 age(years) + b2 age(years)2 + b3 height(cm)2. The coefficients are taken from Tables 4 and 5 of Hankinson *et al.*[18].

For each result not expressed in terms of FEV_1_%P, we selected those NHANES III subjects who had the range of characteristics relevant to that result. These characteristics included the range of the lung function measure provided, age and sex (and in some cases smoking habit or an additional lung function specification). We then applied the FEV_1_ prediction equations to each of the selected subjects and thus estimated the mean value of FEV_1_%P. For example, one study [19] was of males aged 16–74 and gave relative risks for categories of FEV_1_/FVC (<80%, 80-89% and 90%+ of predicted). From the NHANES data we looked within males aged 16–74 and, for each category of FEV_1_/FVC, calculated the mean value of FEV_1_%P. The calculated mean was then used as the dose value for our calculations of β.

One study [20] was a particular problem as the groupings were in terms of residuals from a regression analysis including age, smoking status and current cigarettes smoked. This model was fitted to the NHANES III data, and mean values of FEV_1_%P were calculated for different quartiles of the residuals.

Only one publication [21] provided mean levels for each category when the original measure was FEV_1_%P. Where means were not available, we used the NHANES III dataset to calculate them. This was of particular benefit when dealing with open-ended categories.

#### Predictions and goodness-of-fit of the fitted model

For data presented by grouped levels of FEV_1_%P (or associated measures) the estimate of β was used to calculate predicted RRs and numbers of lung cancer cases at each level corresponding to the observed RRs and numbers. The observed (O) and predicted (P) numbers were then used to derive a chisquared test of goodness-of-fit by summing (O-P)^2^/P, taking the degrees of freedom (d.f) as one less than the number of levels. For defined values of d (0, 0.01-10, 10.01-20, 20.01-30, 30.01-40, >40) O and P were summed over block to similarly derive an overall goodness-of-fit chisquared statistic on 5 d.f. Blocks involving only two levels were ignored for the chisquared tests as providing no useful information on goodness-of-fit.

#### Meta-analysis and meta-regression

Individual study estimates of β and SE β were combined to give overall estimates using inverse-variance weighted regression analysis, equivalent to fixed-effect meta-analysis. Random-effects meta-analyses were also conducted, but are not reported here as the results were virtually identical. Heterogeneity was investigated by testing for significant variation in β, considering the following factors: sex (male, female, combined), publication year (<1990, 1990–1994, 1995+), age at baseline (<50, 50–59, 60+ years), Newcastle-Ottawa quality score (5–7, 8–9), continent (North America, other), mortality or incidence (deaths, incidence, both), population type (general population, other), exposed population (exposed to known lung carcinogens, other), length of follow up (≤15, 16–23, 24+ years), smoking adjustment (yes, no), measure of FEV_1_ reported (FEV_1_%P, other), effect as originally reported (regression coefficient, RR and CI, SMR/SIR) and inverse-variance weight of β (<1000, 1000–2999, 3000+). Simple one factor at a time regressions were carried out first, with the significance of each factor tested by a likelihood-ratio test compared to the null model. A stepwise multiple regression analysis was then carried out to determine which of the factors predicted risk independently.

#### Forest plots

Exp(β) is an estimate of the RR associated with a decrease of 1% in FEV_1_%P. For each such RR included, referenced by the study REF and associated block details such as sex, the RR is shown as a rectangle, the area of which is proportional to its weight. The CI is indicated by a horizontal line. The RRs and CIs are plotted on a logarithmic scale so that the RR is centred in the CI. Also shown are the values of each RR and CI and the weight as a percentage of the total. Results from the meta-analysis are shown at the bottom of the plot. The combined estimate is presented as a diamond, with the width corresponding to the CI and the RR as the centre of the diamond.

#### Publication bias

Publication bias was investigated using Egger’s test [22] and using funnel plots. In the funnel plots, β is plotted against its precision (=1/SE). A dotted vertical line corresponds to the overall estimate.

#### Software

All data entry and most statistical analyses were carried out using ROELEE version 3.1 (available from P.N.Lee Statistics and Computing Ltd, 17 Cedar Road, Sutton, Surrey SM2 5DA, UK). Some analyses were conducted using SAS or Excel 2003.

## Results

### Publications and studies identified

**Figure 1:**
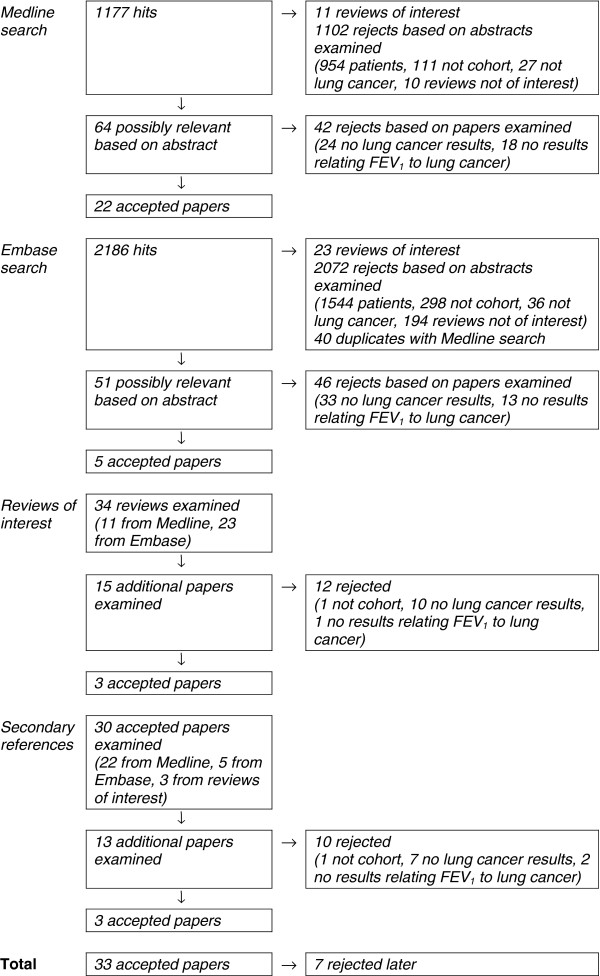
**Flow diagram for literature searching.** The diagram gives details of the four stages of the search; the Medline search, the Embase search, the search based on reviews of interest, and the search based on secondary references. The four criteria for rejecting papers during these four stages are described further in the Methods section under the headings “patients”, “not cohort”, “not lung cancer” and “reviews not of interest”. Note that one of the three papers accepted from the search based on secondary references cited a paper that was also examined but provided no lung cancer results. The four stages produced a total of 33 accepted papers (22 Medline, 5 Embase, 3 reviews of interest, 3 secondary references). Subsequently 7 of these were rejected for reasons described in the first paragraph of the Results section.

Thirty-three publications [1-5,7,9,10,19-21,23-44] satisfying the inclusion and exclusion criteria were identified from the searches carried out in October 2011. Details of these searches are given in Figure 1. Subsequently, at the analysis stage, seven of these publications were rejected. Two [41,42] described a study in Denmark which presented its results in a way that did not allow estimation of β. Two [24,36] described a study in France of iron miners which only provided results for decreased FEV_1_ without giving the ranges of FEV_1_ being compared. One [29] described a nested case–control study in the USA of heavily asbestos-exposed shipyard workers, which reported only the mean difference in FEV_1_ between cases and controls. Two [33,34] described results from the Italian rural cohorts of the Seven Countries Study, which reported results only for forced expiratory volume in ¾ second. A brief summary of the findings from these is reported in Additional file 2: Others, which demonstrates that these were consistent in showing an association of reduced FEV_1_ with increased lung cancer risk. 

**Table 2:** Selected details of the 22 studies of FEV_1_ and lung cancer

**Study REF**	**Reference(s)**	**Location**	**Baseline population**	**Follow-up period (years)**	**Lung cancer cases**	**Newcastle-Ottawa score**^**a**^
BEATY	[23]	USA, Baltimore	874 men aged 17+ entering study on aging between 1958 and 1979	24	15	7
CALABR	[1]	Italy, multicentre	3804 male and female current or former smokers aged 50–75 entering study between 2000 and 2008	5	57	6
CARET	[25,26]	USA, multicentre	3033 male asbestos exposed heavy smokers aged 45–74 entering study between 1985 and 1994	20	205	8
CARTA	[19]	Italy, Sardinia	696 male silicotics aged up to 74 entering study between 1964 and 1970	23	22	6
FINKEL	[27]	Canada, Ontario	733 male radon exposed uranium miners studied in 1974	18	42	5
ISLAM	[4,38]	USA, Michigan	3956 men and women aged 25+ entering community health study between 1962 and 1965	25	77	9
LANGE	[5]	Denmark, Copenhagen	13946 men and women aged 20+ entering heart health study between 1976 and 1978	12	225	8
MALDON	[31]	USA, Minnesota	1520^b^ male and female current or former smokers aged 50+ studied in 1999	4	64	5
MANNIN	[32]	USA, national	5402 men and women aged 25–74 participating in NHANES between 1971 and 1975	22	113	9
MRFIT	[2,30]	USA, multicentre	6613 men aged 35–57 at high risk of heart disease participating in the Multiple Risk Factor Intervention Trial between 1973 and 1982	26	363	8
NOMURA	[7]	USA, Hawaii	6317 Japanese-American men aged 46–68 entering study between 1965 and 1968	22	172	8
PETO	[35]	UK, five areas	2718 men in occupational groups aged 25–64 entering study between 1954 and 1961	25	103	7
PURDUE	[37]	Sweden, national	176997 male construction workers entering study between 1971 and 1993	31	834	7
RENFRE	[3,28]	Scotland, two cities	15244 men and women aged 45–64 entering study between 1972 and 1976	23	651	8
SKILLR	[9]	USA, Minnesota	226^c^ men and women aged 45–59 living in rural areas entering study between 1973 and 1974	11	11	7
SPEIZE	[20]	USA six cities	8427 men and women aged 25–74 entering study between 1974 and 1977	12	61	8
STAVEM	[21]	Norway, Oslo	1623 male workers in five companies aged 40–59 entering study between 1972 and 1975	26	42	7
TAMMEM	[39]	Canada, British Columbia	2596 male and female current and former smokers of 20+ pack-years aged 40+ studied in 1990	17	154	8
TOCKMA	[10]	USA, Baltimore	3728 male current smokers and recent quitters, smoking 1+ packs/day, aged 45+ studied in 1987	2^d^	19	7
VANDEN	[40]	USA, California	153925 male and female members of the Kaiser Permanente Medical Care Program entering study between 1964 and 1972	24	1514	9
WILES	[43]	South Africa, national	2062 male gold miners aged 45–54 entering study between 1968 and 1970	18	74	5
WILSON	[44]	USA, Pennsylvania	1553 male and female current or former smokers of 10+ cigs/day for 25+ years with FEV_1_/FVC <0.7, aged 50–79, entering study from 2002	5	67	6

^a^ See Methods for a description of this score. The maximum possible value is 9.

^b^ Nested case–control analysis involving 64 cases and 377 controls drawn from original population of 1520.

^c^ Nested case–control analysis involving 113 men and women with FEV_1_ <70% predicted, and 113 with FEV_1_ of 85% or more drawn from a study with original sample size not stated.

^d^ Although the mean follow-up was less than 3 years, follow-up for some subjects was 3 years or more, so the study was not considered to have failed the inclusion criteria.

The remaining 26 publications were then subdivided into 22 distinct studies, some details of which are summarized in Table 2. Of the 22 studies, 12 were conducted in the USA, 3 in Scandinavia, 2 in Italy, 2 in the UK, 2 in Canada and 1 in South Africa. Many of the studies were quite old, with 16 starting before 1980. 12 involved follow-up of 20 years or more, with a further 6 involving at least 10 years follow-up. Numbers of lung cancers analysed ranged from 11 in study SKILLR to 1514 in study VANDEN. 10 studies involved over 100 cases. 3 studies involved subjects exposed to known lung carcinogens other than smoking (CARET: asbestos, CARTA: silica, FINKEL: radon) and a further study (WILES) was of gold miners. Newcastle-Ottawa quality scores ranged from 5 to 9, with 10 studies scored as 8 or 9. The 22 studies provided data for 32 independent data blocks, with CARET giving results separately for those with FEV_1_/FVC above or below 0.70, RENFRE, SPEIZE and TAMMEM giving results separately for men and women, ISLAM giving results separately for current and non-current smokers, and VANDEN, the study involving the largest number of lung cancer cases, giving six sets of results, separately for all combinations of sex and smoking status (never, former, current).

**Table 3:** Results for the five blocks already expressed as regression coefficients

**Block: study**	**Block details**	**β (SE)**	**Comment**
**7: ISLAM**	Never and former smokers	0.016 (0.010)	As given (FEV_1_%P)
**8: ISLAM**	Current smokers	0.013 (0.007)	As given (FEV_1_%P)
**10: MALDON**	Whole population	0.015 (0.008)	Given as 1.15 (95% CI 1.00-1.32) for an OR for a 10% decrease in FEV_1_%P
**22: TAMMEM**	Females	0.010 (0.008)	Given as 0.99 (95% CI 0.98-1.01) for an OR for a 1% increase in FEV_1_%P
**23: TAMMEM**	Males	0.030 (0.007)	Given as 0.97 (95% CI 0.96-0.99) for an OR for a 1% increase in FEV_1_%P

### Fitted β estimates and goodness-of-fit

Table 3 summarizes the results for those five blocks where regression estimates for the lung cancer/FEV_1_ relationship were provided by the authors. For two blocks, β was directly available, and for the other three β could readily be calculated from the odds ratio for a given percentage increase or decrease in FEV_1_%P.

**Table 4:** Fit of the model to the data for the 27 blocks with grouped data

**Block: study**^**a**^	**Measure**^**b**^	**Range**^**c**^	**FEV**_1_**%P Diff**^**d**^	**RR (95%CI)**	**Fitted RR**	**Cases observed**^**e**^	**Cases fitted**
**1: BEATY**	FEV_1_%P	>80	(95.33)	1.00	1.00	14.30	14.30
β (SE) =	−0.028 (0.034)	≤80	29.77	0.43 (0.06-3.20)	0.43	1.03	1.03
**2: CALABR**	FEV_1_%P	90+	(104.94)	1.00	1.00	24.20	25.71
β (SE) =	0.024 (0.008)	70 to <90	23.90	2.29 (1.24-4.23)	1.76	17.09	13.98
*χ*^2^ (df) =	1.04 (1)	<70	49.60	2.90 (1.34-6.27)	3.25	8.50	10.11
**3: CARET**^**f**^	FEV_1_%P	80+	(100.75)	1.00	1.00	35.35	34.69
β (SE) =	0.022 (0.007)	70 to <80	24.89	1.54 (0.80-2.63)	1.74	14.59	16.20
*χ*^2^ (df) =	0.22 (2)	60 to <70	34.92	2.25 (1.20-4.19)	2.18	12.39	11.77
		<60	49.07	3.08 (1.42-6.69)	2.99	6.92	6.59
**4: CARET**^**g**^	FEV_1_%P	80+	(91.97)	1.00	1.00	16.66	15.78
β (SE) =	0.012 (0.006)	70 to <80	16.94	1.05 (0.56-1.96)	1.22	19.07	20.99
*χ*^2^ (df) =	0.25 (2)	60 to <70	26.77	1.33 (0.74-2.42)	1.36	24.04	23.27
		<60	47.77	1.66 (0.95-2.89)	1.74	34.62	34.35
**5: CARTA**	FEV_1_/FVC	90+	(99.82)	1.00	1.00	3.72	7.84
β (SE) =	0.072 (0.049)	80 to <90	−0.99	3.87 (1.12-15.05)	0.93	5.83	2.95
*χ*^2^ (df) =	5.25 (1), p<0.05	<80	9.64	5.18 (1.56-19.66)	2.00	6.66	5.42
**6: FINKEL**	FEV_1_%P	100+	(109.54)	1.00	1.00	7.75	6.71
β (SE) =	0.009 (0.011)	80 to <100	18.00	0.89 (0.39-2.18)	1.17	13.43	15.32
*χ*^2^ (df) =	0.47 (1)	<80	41.17	1.35 (0.57-3.36)	1.44	11.17	10.31
**9: LANGE**	FEV_1_%P	80+	(100.99)	1.00	1.00	47.92	48.77
β (SE) =	0.020 (0.004)	40 to <80	32.64	2.10 (1.30-3.40)	1.93	24.67	23.05
*χ*^2^ (df) =	0.17 (1)	<40	69.50	3.90 (2.20-7.20)	4.05	13.46	14.23
**11: MANNIN**	FEV_1_%P	80+	(100.24)	1.00	1.00	84.98	84.94
β (SE) =	0.022 (0.006)	<80	33.97	2.12 (1.44-3.11)	2.12	35.83	35.87
**12: MRFIT**	FEV_1_ unnormalised,ml	≥3674	(105.91)	1.00	1.00	27.01	26.50
β (SE) =	0.031 (0.005)	3307 to 3673	10.05	1.31 (0.82-2.10)	1.37	45.30	46.34
*χ*^2^ (df) =	0.85 (3)	2985 to 3306	15.92	1.50 (0.95-2.36)	1.64	54.20	58.27
		2606 to 2984	22.21	2.13 (1.39-3.26)	2.00	80.62	74.45
		≤2605	37.59	3.13 (2.07-4.72)	3.23	106.01	107.59
**13: NOMURA**	FEV_1_%P	103.5+	(113.14)	1.00	1.00	22.16	23.76
β (SE) =	0.018 (0.005)	94.5 to <103.5	14.40	1.00 (0.60-1.90)	1.29	23.34	32.35
*χ*^2^ (df) =	11.40 (2), p<0.01	84.5 to <94.5	23.45	2.50 (1.50-4.10)	1.52	44.66	29.09
		<84.5	43.11	2.10 (1.30-3.50)	2.15	49.51	54.48
**14: PETO**	SDs of FEV_1_/h^3^ below average	Above average	(103.85)	1.00	1.00	32.15	39.80
β (SE) =	0.018 (0.008)	0 to 1	15.05	2.17 (1.40-3.38)	1.32	46.77	35.18
*χ*^2^ (df) =	6.81 (2), p<0.05	1 to 2	34.30	2.02 (0.97-3.90)	1.88	9.93	11.43
		2+	65.85	1.89 (0.37-5.90)	3.35	2.03	4.47
**15: PURDUE**	FEV_1_%P	80+	(100.13)	1.00	1.00	1698.83	1698.83
β (SE) =	0.023 (0.002)	<80	31.76	2.06 (1.77-2.39)	2.06	189.24	189.24
**16: RENFRE**^**h**^	FEV_1_%P	Quintile 5	(116.04)	1.00	1.00	31.54	35.64
β (SE) =	0.015 (0.003)	Quintile 4	13.70	1.36 (0.86-2.13)	1.22	42.34	42.97
*χ*^2^ (df) =	1.48 (3)	Quintile 3	23.79	1.81 (1.18-2.78)	1.42	55.91	49.41
		Quintile 2	35.19	1.93 (1.27-2.94)	1.67	62.88	61.57
		Quintile 1	57.75	2.53 (1.69-3.79)	2.32	79.83	82.90
**17: RENFRE**^**i**^	FEV_1_%P	Quintile 5	(119.96)	1.00	1.00	6.22	15.69
β (SE) =	0.011 (0.005)	Quintile 4	13.53	3.63 (1.49-8.84)	1.15	21.19	16.98
*χ*^2^ (df) =	8.39 (3), p<0.05	Quintile 3	24.26	4.03 (1.68-9.67)	1.29	24.88	20.11
		Quintile 2	36.19	4.12 (1.73-9.81)	1.47	27.21	24.40
		Quintile 1	59.75	4.37 (1.84-10.42)	1.88	27.40	29.72
**18: SKILLR**	FEV_1_%P	85+	(100.81)	1.00	1.00	1.99	1.99
β (SE) =	0.034 (0.017)	< = 70	44.85	4.50 (0.99-20.37)	4.50	8.97	8.97
**19: SPEIZE**^**h**^	Mean FEV_1_,ℓ.^j^	4.07	(109.98)	1.00	1.00	2.09	3.72
β (SE) =	0.048 (0.014)	3.54	9.02	4.33 (1.19-23.71)	1.54	9.35	5.93
*χ*^2^ (df) =	4.49 (2)	3.18	16.84	2.10 (0.45-12.96)	2.25	3.85	7.35
		2.55	33.30	9.60 (2.93-49.67)	4.98	21.90	20.19
**20: SPEIZE**^**i**^	Mean FEV_1_,ℓ.^j^	2.90	(112.16)	1.00	1.00	0.50	0.62
β (SE) =	0.054 (0.029)	2.57	10.10	3.17 (0.25-166.25)	1.73	1.36	0.92
*χ*^2^ (df) =	0.52 (2)	2.34	19.47	2.05 (0.11-121.19)	2.86	0.85	1.48
		1.95	35.49	8.94 (1.20-396.75)	6.80	5.74	5.43
**21: STAVEM**	Mean FEV_1_%P	121.90	(121.90)	1.00	1.00	8.99	6.19
β (SE) =	0.021 (0.008)	106.60	15.30	0.78 (0.29-2.07)	1.38	6.89	8.42
*χ*^2^ (df) =	4.48 (2)	95.30	26.60	0.67 (0.24-1.86)	1.76	6.00	10.86
		75.70	46.20	2.23 (1.03-4.83)	2.67	20.40	16.82
**24: TOCKMA**	FEV_1_%P	>85	(102.90)	1.00	1.00	22.27	22.72
β (SE) =	0.021 (0.010)	60 to 85	27.52	2.57 (0.87-7.56)	1.78	3.82	2.70
*χ*^2^ (df) =	0.60 (1)	<60	57.43	2.72 (0.76-9.74)	3.34	2.61	3.27
**25: VANDEN**^**k**^	FEV_1_ unnormalised, ℓ	3.85+	(105.38)	1.00	1.00	5.34	4.12
β (SE) =	0.018 (0.013)	3.35-3.85	7.12	1.19 (0.41-3.49)	1.14	8.82	6.47
*χ*^2^ (df) =	2.23 (3)	2.85-3.35	9.72	0.76 (0.27-2.14)	1.19	10.80	13.02
		2.35-2.85	11.23	0.76 (0.27-2.14)	1.22	11.01	13.64
		<2.35	31.46	1.49 (0.55-4.05)	1.75	13.75	12.46
**26: VANDEN**^**l**^	FEV_1_ unnormalised, ℓ	3.85+	(106.21)	1.00	1.00	6.84	10.05
β (SE) =	0.010 (0.007)	3.35-3.85	5.47	1.43 (0.60-3.42)	1.06	18.46	20.11
*χ*^2^ (df) =	1.63 (3)	2.85-3.35	10.13	1.76 (0.78-3.93)	1.11	41.81	38.92
		2.35-2.85	14.67	1.80 (0.83-3.91)	1.17	81.93	78.13
		<2.35	35.27	2.04 (0.92-4.54)	1.44	45.94	47.77
**27: VANDEN**^**m**^	FEV_1_ unnormalised, ℓ	3.85+	(104.52)	1.00	1.00	24.68	29.86
β (SE) =	0.012 (0.003)	3.35-3.85	9.72	1.32 (0.82-2.12)	1.12	55.20	56.66
*χ*^2^ (df) =	1.69 (3)	2.85-3.35	14.29	1.43 (0.94-2.19)	1.18	141.35	140.99
		2.35-2.85	21.15	1.62 (1.08-2.44)	1.28	267.93	255.94
		<2.35	40.79	1.89 (1.24-2.87)	1.61	167.40	173.12
**28: VANDEN**^**n**^	FEV_1_ unnormalised, ℓ	2.75+	(105.01)	1.00	1.00	7.50	5.82
β (SE) =	−0.004 (0.016)	2.35-2.75	7.32	0.76 (0.30-1.90)	0.97	11.34	11.31
*χ*^2^ (df) =	2.92 (3)	2.05-2.35	8.70	0.60 (0.27-1.34)	0.97	29.24	36.34
		1.65-2.05	8.73	0.92 (0.40-2.12)	0.97	21.57	17.51
		<1.65	22.96	0.76 (0.33-1.78)	0.91	18.43	17.11
**29: VANDEN**^**o**^	FEV_1_ unnormalised, ℓ	2.35-2.75^p^	(97.83)	1.00	1.00	11.85	9.95
β (SE) =	0.026 (0.011)	2.05-2.35	3.02	1.25 (0.61-2.57)	1.08	19.20	13.93
*χ*^2^ (df) =	6.55 (2), p<0.05	1.65-2.05	5.49	0.54 (0.24-1.21)	1.15	11.29	20.27
		<1.65	27.83	1.92 (0.92-4.02)	2.05	17.24	15.43
**30: VANDEN**^**q**^	FEV_1_ unnormalised, ℓ	2.75+	(103.28)	1.00	1.00	9.63	21.54
β (SE) =	0.019 (0.004)	2.35-2.75	9.74	2.90 (1.46-5.77)	1.20	51.91	47.91
*χ*^2^ (df) =	8.13 (3), p<0.05	2.05-2.35	15.25	3.33 (1.72-6.48)	1.33	86.50	77.06
		1.65-2.05	21.26	3.33 (1.74-6.37)	1.48	166.89	166.21
		<1.65	41.80	4.76 (2.47-9.19)	2.17	107.33	109.53
**31: WILES**	FEV_1_/h^3^, cl/m^3^	56+	(105.56)	1.00	1.00	23.36	25.12
β (SE) =	0.021 (0.008)	43-56	17.66	1.69 (0.97-2.94)	1.46	25.01	23.20
*χ*^2^ (df) =	1.02 (2)	30-43	36.24	2.65 (1.29-5.20)	2.17	11.02	9.68
		0-30	70.93	2.87 (0.56-9.30)	4.54	1.97	3.34
**32: WILSON**^**g**^	FEV_1_%P	80+	(100.14)	1.00	1.00	10.78	10.85
β (SE) =	0.008 (0.007)	50 to <80	30.94	1.30 (0.64-2.65)	1.28	22.87	22.73
*χ*^2^ (df) =	0.002 (1)	<50	62.07	1.65 (0.70-3.90)	1.65	9.21	9.28
**TOTAL**^r^	FEV_1_%P		(106.16)			388.51	431.45
*χ*^2^ (df) =	8.43 (5)		0.01-10			259.67	257.83
			10.01-20			666.20	658.51
			20.01-30			742.1	694.76
			30.01-40			364.31	358.07
			>40			542.36	562.52

^a^ For each block, the block number and study reference code is shown. Also shown in columns 1 and 2 are the values of β, the fitted slope of the relationship of log RR to the estimated mean difference (see note d), and the SE of β, and also, for blocks with more than two levels, the results of the goodness-of-fit test.

^b^ This is the measure the data were originally recorded in.

^c^ The range of values of the measure for which results were available.

^d^ The estimated mean difference of FEV_1_%P between the comparison level and the level of interest. Shown in brackets is the estimate of FEV_1_%P for the comparison level.

^e^ These are pseudo-numbers of cases estimated using the method of Hamling *et al.*[16].

^f^ FEV_1_/FVC ≥0.70.

^g^ FEV_1_/FVC<0.70.

^h^ Males.

^i^ Females.

^j^ RRs were given by quartiles of FEV_1_ residuals calculated from a prediction equation. Mean FEV_1_ levels for each quartile were used to derive the differences in FEV_1_%P.

^k^ Male never smokers.

^l^ Male former smokers.

^m^ Male current smokers.

^n^ Female never smokers.

^o^ Female former smokers.

^p^ There were no deaths in the highest quintile (2.75+ ℓ).

^q^ Female current smokers.

^r^ Total over all blocks with more than two levels.

Table 4 summarizes the results for the remaining 27 blocks where results were given by level of FEV_1_%P or an associated measure. The table shows the measure the data were originally presented in, the estimated mean reduction in FEV_1_%P compared to the base group with the highest value of FEV_1_%P, the observed RRs and 95% CIs and those fitted using the estimate of β, which is also shown. Also shown are the observed pseudo-numbers of lung cancer cases at each level and those fitted using the estimate of β, and the goodness-of-fit chisquared. Additional file 3: Fit gives plots comparing the observed and fitted RRs.

**Table 5:** Testing for significance of variation in β by various factors considered one at a time

**Factor**	**Level**	**Blocks included**^**a**^	**N**^**b**^	**β (95% CI)**	**Deviance**^**c**^
None	All	1-32	32	0.018 (0.016-0.021)	44.01
Sex	Male	1,3-6,12-16,19,21,23-27,31	18	0.019 (0.016-0.022)	42.33
	Female	17,20,22,28-30	6	0.015 (0.008-0.022)	
	Both	2,7-11,18,32	8	0.018 (0.012-0.024)	
Publication year	<1990	1,14,18-20,24	6	0.025 (0.012-0.038)	40.12
	1990-1994	5,7-9,13,25-31	12	0.016 (0.012-0.020)	
	1995+	2-4,6,10-12,15-17,21-23,32	14	0.019 (0.016-0.023)	
Mean age	<50	5,6,11,12,14,15,19-21,31	10	0.024 (0.020-0.028)	29.12**
	50-59	1,3,4,9,13,16-18,25-30	14	0.015 (0.012-0.018)	
	60+	2,7,8,10,22-24,32	8	0.017 (0.011-0.022)	
Quality score	8 or 9	3,4,7-9,11-13,16,17,19,20,22,23, 25–30,32	21	0.017 (0.014-0.020)	40.20
	5 to 7	1,2,5,6,10,14,15,18,21,24,31	11	0.022 (0.017-0.026)	
Continent	North America	1,3,4,6-8,10-13,18-20,22-30,32	23	0.018 (0.014-0.021)	43.46
	Other	2,5,9,14-17,21,31	9	0.019 (0.016-0.023)	
Disease fatality	Deaths	1,5,9,12,14,15,19-21,24,31	11	0.024 (0.020-0.027)	28.99**
	Incidence	13,22,23,25-30	9	0.015 (0.011-0.020)	
	Both	2-4,6-8,10,11,16-18,32	12	0.015 (0.012-0.019)	
Population type	General	1,7-9,11,14,16,17,19-21,25-30	17	0.016 (0.013-0.019)	37.74*
	Other	2-6,10,12,13,15,18,22-24,31,32	15	0.021 (0.018-0.025)	
Exposed to lung carcinogens	Yes	3-6	4	0.016 (0.006-0.025)	43.44
	No	1,2,7-32	28	0.019 (0.016-0.021)	
Follow-up period	1-15	2,9,10,18-20,24,32	8	0.020 (0.013-0.027)	41.72
	16-23	3-6,11,13,16,17,22,23,31	11	0.016 (0.012-0.021)	
	24+	1,7,8,12,14,15,21,25-30	13	0.019 (0.016-0.023)	
Adjusted for smoking	Yes^d^	2-4,7-13,15-17,19,20,22-30,32	25	0.018 (0.016-0.021)	43.98
	No	1,5,6,14,18,21,31	7	0.019 (0.009-0.029)	
Measure of FEV1 reported	FEV_1_%P	1-4,6-11,13,15-18,21-24,32	20	0.018 (0.015-0.021)	43.93
	Other	5,12,14,19,20,25-31	12	0.019 (0.014-0.024)	
Weight of β	<125	1,5-7,18-20,24,25,28,29	11	0.021 (0.010-0.031)	43.54
	125-250	2,3,8,10,14,21-23,26,31,32	11	0.017 (0.012-0.023)	
	250+	4,9,11-13,15-17,27,30	10	0.019 (0.015-0.022)	
Original data recorded as	Regression coefficient	7,8,10,22,23	5	0.017 (0.008-0.026)	43.02
	RR (CI)	1-4,9,11-13,15-18,21,24-30,32	21	0.018 (0.016-0.021)	
	SMR/SIR	5,6,14,19,20,31	6	0.022 (0.011-0.034)	

^a^ See Tables 3 and 4 for definition of blocks.

^b^ Number of estimates of β which are combined.

^c^ The significance of the factor is assessed by comparing the deviance for the model including that factor and the deviance for the null (no factor) model and is indicated by *p<0.05 **p<0.01 *** p<0.001.

^d^ This includes blocks which relate to the whole population, current smokers or ever smokers which adjust at least for a measure of dose, such as cigs/day or pack yrs, and blocks which are restricted to nonsmokers.

Where only two levels of FEV_1_%P were available, the fitted numbers of cases necessarily equalled the numbers observed. Where there were more than two levels being compared, the goodness-of-fit to the model was generally satisfactory. The significant (p<0.05) misfits to the model were for: block 5 (CARTA), where there was almost a 4-fold difference in risk between the highest and middle groups (90+ and 80 to <90 FEV_1_/FVC) but virtually the same estimated FEV_1_%P; block 13 (NOMURA) and block 29 (VANDEN female former smokers), where the pattern of increasing risk with declining FEV_1_%P was non-monotonic; and block 14 (PETO), block 17 (RENFRE females) and block 30 (VANDEN female current smokers), where the increase in risk was similar but marked in all the groups with reduced FEV_1_%P. Only for block 13 (NOMURA) was the p value for the fit <0.01. Table 4 also includes the results from an overall goodness-of-fit test for those blocks involving more than two levels. While there is some tendency for fitted numbers of lung cancer cases to be somewhat higher than the observed numbers at the extremes (the comparison group and differences in FEV_1_%P greater than 40), and lower in the four intermediate groups (differences of 0.01 to 10, 10.01 to 20, 20.01 to 30 and 30.01 to 40) the goodness-of-fit chisquared statistic of 8.43 on 5 d.f. is not significant (p=0.13).

### Meta-analysis and meta-regressions

**Figure 2:**
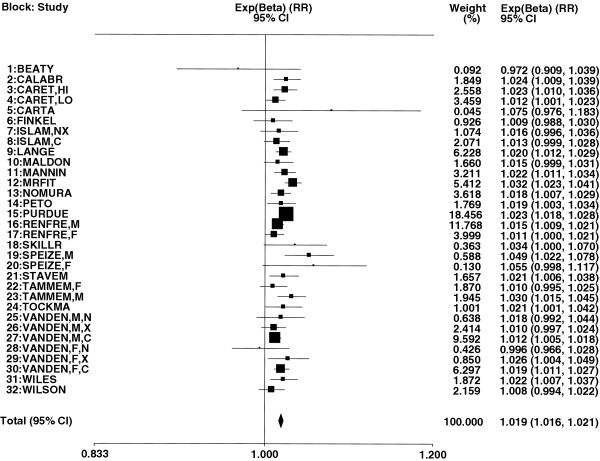
**Forest plot of the 32 estimates of exp(β).** Estimates of β and SE(β) are presented in Table 3 for results presented originally as regression coefficients and in Table 4 for results presented by grouped level of FEV1 or associated measures. For each of the 32 estimates Figure 2 shows the associated values of exp(β) with their 95%CIs. These estimates are shown both numerically and also graphically on a logarithmic scale. The studies are sorted in order of block number, and are referenced by study reference (REF). Multiple blocks within the same study are distinguished by the following codes (M = males, F = females, N = never smokers, X = ex smokers, C = current smokers, LO = FEV_1_/FVC ≥ 0.70, and HI = FEV_1_/FVC < 0.70). In the graphical representation individual RRs are indicated by a solid square, with the area of the square proportional to the weight (inverse- variance of log RR).

Exp(β) is the RR associated with a decrease in FEV_1_%P by one unit, and Figure 2 presents a forest plot showing the estimated values with 95% CI for each of the 32 blocks. These range from 0.972 to 1.075, with a combined estimate of 1.019 (95% CI 1.016 to 1.021, p<0.001). It is evident from Figure 2 that the estimates are reasonably consistent. As shown in Table 5, the deviance (chisquared) of the 32 results is 44.01 on 31 d.f., equivalent to an I^2^ of 29.6%.

Table 5 also presents estimates of β by level of a range of different factors. For 10 of the 13 factors considered, including sex, publication year, study quality, continent, exposed to lung carcinogens, follow-up period, smoking adjustment, measure of FEV_1_ reported, inverse-variance weight of β, and how the data were originally recorded, there was no significant evidence of variation by level. However, there was significant evidence of variation by mean age at baseline (p<0.01), disease fatality (p<0.01) and population type (p<0.05), with estimates of β being somewhat higher in younger populations, in studies involving lung cancer deaths rather than incidence, and in studies not of the general population. In stepwise regression, however, only mean age at baseline remained in the model as an independent predictor of lung cancer risk.

### Publication bias

**Figure 3:**
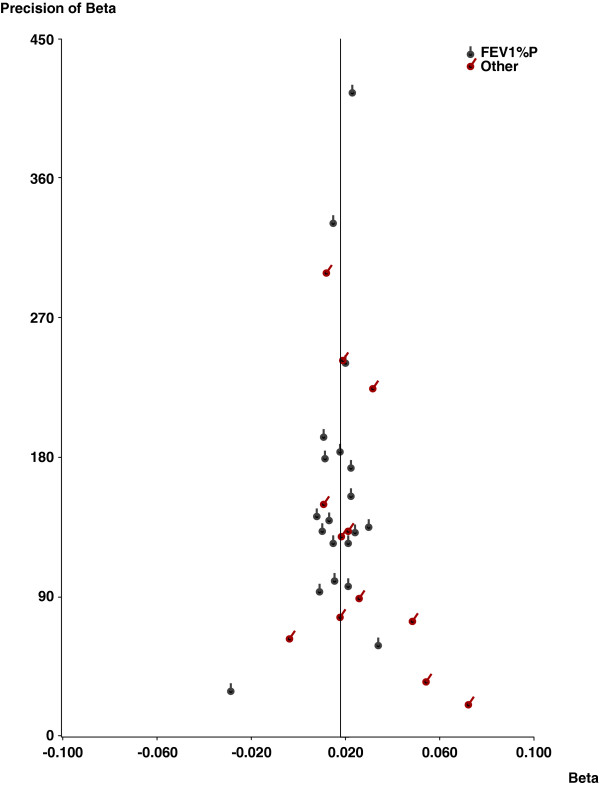
**Funnel plot.** Funnel plot of the 32 estimates of β against their precision (1/SE). The dotted vertical line indicates the meta-analysis estimate. Estimates based on data originally presented as FEV_1_%P are distinguished from other estimates by different symbols.

Based on the 32 estimates of β there was no evidence of publication bias using Egger’s test. This is consistent with the funnel plot shown as Figure 3, and with the lack of relationship between β and its weight shown in Table 5.

## Discussion

Based on 32 independent data sets from 22 studies we estimate β as 0.018 (95%CI 0.016-0.021). This relationship is highly significant (p<0.001) and is equivalent to saying that, compared to someone with an average FEV_1_%P of 100%, someone with an FEV_1_%P of 90% would have a 20% increase in lung cancer risk, and someone with an FEV_1_%P of 50% would have a 151% increase.

There is little evidence of heterogeneity over study (I^2^ = 29.6%), or that estimates vary by specific factors including sex, study location, length of follow-up, adjustment for smoking, the measure of FEV_1_ reported, or how the results were originally reported. Nor was there any evidence of publication bias. There was, however, some evidence that estimates varied by age of the population at baseline, but even then clear reductions were seen in all three age groups studied, with β varying only between 0.015 and 0.024. We discuss below various aspects of our methods, which might attract criticism.

One is the use of the data from NHANES III which, though nationally representative of the USA, would not be representative of the populations involved in the 22 studies we considered. We used NHANES III for two reasons. First, we needed to have mean FEV_1_%P values corresponding to the groups used, only one study actually reported such means, and NHANES III was a large and available database. Our feeling is that any errors for non open-ended intervals are likely to be minor, and that even for open-ended intervals any errors are unlikely to have affected our main conclusions. In this we are fortified by the general consistency of the estimates of β and also by the observation that for the one study (STAVEM) that did supply means, the estimates reported (121.9, 106.6, 95.3 and 75.7) were similar to those that could be estimated from NHANES III (122.1, 106.2, 94.8 and 71.9). The other reason was that we needed some method of incorporating studies reporting results, not by FEV_1_%P directly, but by associated measures. Had we restricted attention to results reported by FEV_1_%P we would have reduced the number of available blocks from 32 to 20, and we wished to avoid such loss of power. Here it is reassuring that the overall estimate for the 12 blocks where β was estimated using data for associated measures of 0.019 (0.014-0.024) was very close to that for the other 20 blocks of 0.018 (0.015-0.021).

We should also comment on the fact that the method of estimation of β required pseudo-numbers of cases and numbers at risk for each level of FEV_1_%P corresponding to the adjusted RRs, as using simple numbers would have removed the effects of adjustment. We used the method of Hamling *et al.*[16] here to estimate the pseudo-numbers, and note that Orsini *et al.*[45] recently reported that they arrived at very similar results using this method as they obtained based on the available individual person data, although this was in a somewhat different context. Our experience too is that the method provides a very robust way of estimating the magnitude and significance of functions of relative risks. 

Another issue is the use of a simple model in which the logarithm of the RR is linearly related to the difference in FEV_1_%P. As always, one could postulate more complex relationships, but have found that the model fits the data quite well, as judged by the goodness-of-fit tests conducted. We have not explored whether more complex models fit materially better, nor attempted to estimate risks for a given level of FEV_1_%P, but note that a simple model has advantages in expressing the relationship to the reader. Clearly our model may not fit perfectly at the extremes (e.g. comparing someone with a value of FEV_1_%P of 150 and one of 30) but data here are limited. One would really need individual person data to get a more precise answer, but we have not attempted to obtain such data, particularly as many of the studies were conducted many years ago.

Based on those studies where we could estimate β we found no evidence of publication bias. However, we should point out that we had to reject seven publications, describing four studies, as the data were not presented in a way that allowed estimation of β. These studies, which each involved less than 40 lung cancer cases, were consistent in demonstrating a positive association of reduced FEV_1_ with increased lung cancer risk, and it seems unlikely that this omission has caused material bias.

While our β estimates were quite consistent over study, we did observe somewhat higher values in younger populations. This may reflect variations in the rate of FEV_1_ decline associated with susceptibility to smoking [46]. Subjects in younger populations who already have reduced FEV_1_ may have even more reduced FEV_1_ later in life and therefore an even greater risk of lung cancer during follow-up. None of the studies we reviewed relate FEV_1_ recorded on two occasions to subsequent risk of lung cancer, to allow direct testing of the relationship of rapidity of FEV_1_ decline to lung cancer risk.

In their review Wasswa-Kintu *et al.*[11] concluded that “reduced FEV_1_ is strongly associated with lung cancer” and that “even a relatively modest reduction in FEV_1_ is a significant predictor of lung cancer, especially among women.” Their meta-analyses were based on four studies that reported FEV_1_ in quintiles, with their estimated relative risks for the lowest to the highest quintile being 2.23 (95%CI 1.73-2.86) for men and 3.97 (95%CI 1.93-8.25) for women. While our meta-analyses, which are based on far more studies, confirmed the strong association of reduced FEV_1_ with increased lung cancer risk, we found no significant difference between the sexes. It is not possible to compare our estimates precisely but, taking the difference in FEV_1_%P between the lowest and highest quintiles to be 60 (approximately the value for the NHANES III population for both sexes), our estimate of β of 0.0184 predicts a lowest to highest quintile relative risk of 3.02, which is not very different from the estimates of Wasswa-Kintu *et al.*[11].

## Conclusions

Our review confirms the strong association between reduced FEV_1_ and increased risk of lung cancer. The strength of the association is very consistent, with our 32 estimates of β showing remarkably little variation, given the variety of ways in which the source papers presented their results. Based on our results, we estimate that each 10% decrease in FEV_1_%P is associated with a 20% (95% CI 17%-23%) increase in lung cancer risk.

## Abbreviations

CI: Confidence Interval; d.f.: Degrees of Freedom; FEV_1_: Forced Expiratory Volume in 1 second; FEV_1_%P: FEV_1_ expressed as a percentage of predicted; FVC: Forced Vital Capacity; NHANES: National Health and Nutrition Examination Surveys; REF: 6 character Reference code used to identify a study; RR: Relative Risk; SE: Standard error.

## Competing interests

PNL, founder of P.N.Lee Statistics and Computing Ltd., is an independent consultant in statistics and an advisor in the fields of epidemiology and toxicology to a number of tobacco, pharmaceutical and chemical companies. This includes Philip Morris Products S.A., the sponsor of this study. JSF and JSH are employees of P.N.Lee Statistics and Computing Ltd.

## Authors’ contributions

JSF and PNL were responsible for planning the study. Literature searches were carried out by PNL and KJC. Data entry was carried out by JSH and checked by PNL or JSF. The statistical analyses were conducted by JSF along lines discussed and agreed with PNL. PNL drafted the paper, which was then critically reviewed by JSF and JSH. All authors read and approved the final manuscript.

## Pre-publication history

The pre-publication history for this paper can be accessed here:

http://www.biomedcentral.com/1471-2407/12/498/prepub

## Supplementary Material

Additional file 1 **Quality.** DOC file which describes the components of the Newcastle-Ottawa study quality scoring system, shows the scores allocated to each study, and for some scores gives the reason the study scored as negative. Scores relate to eight items - 1: “*representativeness of the exposed cohort*”, 2: “*selection of the non-exposed cohort*”, 3: “*ascertainment of exposure*”, 4: “*demonstration that the outcome of interest was not present at start of the study*”, 5: “*comparability of the cohorts on the basis of design or analysis*”, 6: “*assessment of outcome*”, 7: “*was follow-up long enough for outcomes to occur”,* and 8: “*adequacy of follow up of cohorts*”. Apart from item 5, which is scored as 0, 1 or 2, each item is scored as 0 or 1, so the total possible score for a study is 9. Click here for file

Additional file 2 **Others.** DOC file summarizes the results for the four studies which satisfied the inclusion/exclusion criteria but were later rejected as estimates of β could not be derived. Click here for file

Additional file 3 **Fit.** DOC file giving, for each of the blocks considered in Table 4 that include more than two levels, a plot by decline in FEV_1_%P of the observed RRs (with 95% CIs) and the RRs fitted based on the value of β for that block. The fitted value of β and its SE are shown in the heading for the block. Click here for file
