# Supplementary material for: Systematic review with meta-analysis of the epidemiological evidence relating FEV1 decline to lung cancer risk
Source: BMC Cancer. 2012 Oct 27;12:498. doi: 10.1186/1471-2407-12-498 (PMC3573968; doi:10.1186/1471-2407-12-498)
Supplement: Additional file 1 — Quality. DOC file which describes the components of the Newcastle-Ottawa study quality scoring system, shows the scores allocated to each study, and for some scores gives the reason the study scored as negative. Scores relate to eight items - 1: “representativeness of the exposed cohort”, 2: “selection of the non-exposed cohort”, 3: “ascertainment of exposure”, 4: “demonstration that the outcome of interest was not present at start of the study”, 5: “comparability of the cohorts on the basis of design or analysis”, 6: “assessment of outcome”, 7: “was follow-up long enough for outcomes to occur”, and 8: “adequacy of follow up of cohorts”. Apart from item 5, which is scored as 0, 1 or 2, each item is scored as 0 or 1, so the total possible score for a study is 9. [file 1471-2407-12-498-S1.doc]

# Additional file 1: Quality

The table below shows the scores allocated to each study using the Newcastle-Ottawa scoring scale, and for some scores the reason the study scored as negative.

The first score, “*representativeness of the exposed cohort*”, is scored as positive where the study was considered truly or somewhat representative of the general population in the location of the study. Studies on selected groups or where there was no description if the cohort were scored as negative.

The second score, “*selection of the non-exposed cohort*”, is scored as positive for all the studies because, for each study, the population was selected and then each subject was identified as either exposed or non-exposed, so that the non-exposed cohort was drawn from the same community as the exposed cohort.

The third score, “*ascertainment of exposure*”, in our analysis, relates to measurement of FEV_1_. For each study this was done using spirometry, and so comes from secure records.

The fourth score, “*demonstration that the outcome of interest was not present at start of the study*”, is scored as positive if it is stated that the subjects were free of lung cancer or were healthy, or that they underwent a chest x-ray or other chest scan.

The fifth score, “*comparability of the cohorts on the basis of design or analysis*”, was scored according to whether the analysis controlled for smoking and/or for an additional factor. Age adjustment was not considered in this assessment, as this was considered fundamental. The scores relate to the analyses used in our study so, for example, if the source paper presented some adjusted analyses but the analysis of FEV_1_ and lung cancer was unadjusted, the study is scored negatively.

The sixth score, “*assessment of outcome*”, was scored positively if record linkage and/or death certificates or an independent assessment was used to identify the subjects who developed lung cancer.

The seventh score is “*was follow-up long enough for outcomes to occur”*. It was decided that a follow-up of less than 5 years is not really adequate.

The eighth and final score, “*adequacy of follow up of cohorts*” was scored positively if the follow-up was complete or the subjects lost to follow up were unlikely to introduce bias. The great majority of the studies had virtually complete follow-up of study participants through linkage to cancer registries and death records or through continued contact with study subjects and their families.

The highest possible total score is 9. The distribution of total scores in the 22 studies is as follows: 5 (3 studies), 6 (2 studies), 7 (6 studies), 8 (8 studies) and 9 (3 studies). Splitting the studies into the 11 better studies with scores of 8 or 9, and the 11 worse studies with scores of 5, 6 or 7 seems appropriate for testing for effects of study quality.

| Study blocks and  references | | Represent-ativeness  of the exposed cohort | Selection of the non-exposed cohort | Ascertainment of exposure:  FEV_1_ measurement | Demonstration that lung cancer was not present at the start | Study controls for smoking and/or an additional factor | Assessment of outcome | Was follow-up  5 years or more? | Adequacy of follow-up | Total |
| --- | --- | --- | --- | --- | --- | --- | --- | --- | --- | --- |
| 1 | BEATY | ★ | ★ | ★ | ★ | — | ★ | ★ | ★ | 7 |
| 2 | CALABR | —(a) | ★ | ★ | ★ | ★★ | —(f) | ★ | —(f) | 6 |
| 3,4 | CARET | —(b) | ★ | ★ | ★ | ★★ | ★ | ★ | ★ | 8 |
| 5 | CARTA | —(b) | ★ | ★ | ★ | — | ★ | ★ | ★ | 6 |
| 6 | FINKEL | —(b) | ★ | ★ | ★ | — | ★ | ★ | —(h) | 5 |
| 7,8 | ISLAM | ★ | ★ | ★ | ★ | ★★ | ★ | ★ | ★ | 9 |
| 9 | LANGE | ★ | ★ | ★ | — | ★★ | ★ | ★ | ★ | 8 |
| 10 | MALDON | —(a) | ★ | ★ | ★ | ★ (d) | —(g) | — | ★ | 5 |
| 11 | MANNIN | ★ | ★ | ★ | ★ | ★★ | ★ | ★ | ★ | 9 |
| 12 | MRFIT | —(a) | ★ | ★ | ★ | ★★ | ★ | ★ | ★ | 8 |
| 13 | NOMURA | —(c) | ★ | ★ | ★ | ★★ | ★ | ★ | ★ | 8 |
| 14 | PETO | ★ | ★ | ★ | — | ★ (e) | ★ | ★ | ★ | 7 |
| 15 | PURDUE | —(b) | ★ | ★ | — | ★★ | ★ | ★ | ★ | 7 |
| 16,17 | RENFRE | ★ | ★ | ★ | — | ★★ | ★ | ★ | ★ | 8 |
| 18 | SKILLR | ★ | ★ | ★ | ★ | — | ★ | ★ | ★ | 7 |
| 19,20 | SPEIZE | ★ | ★ | ★ | — | ★★ | ★ | ★ | ★ | 8 |
| 21 | STAVEM | ★ | ★ | ★ | ★ | — | ★ | ★ | ★ | 7 |
| 22,23 | TAMMEM | —(a) | ★ | ★ | ★ | ★★ | ★ | ★ | ★ | 8 |
| 24 | TOCKMA | —(a) | ★ | ★ | ★ | ★★ | ★ | — | ★ | 7 |
| 25-30 | VANDEN | ★ | ★ | ★ | ★ | ★★ | ★ | ★ | ★ | 9 |
| 31 | WILES | —(b) | ★ | ★ | — | — | ★ | ★ | ★ | 5 |
| 32 | WILSON | —(a) | ★ | ★ | ★ | ★★ | ★ | ★ | ★ | 8 |

a. Study restricted by smoking habit and did not include never smokers

b. Study of occupational groups

c. Study of a racial group

d. Adjusted only for smoking

e. Adjusted only for a factor other than smoking

f. No information provided

g. Although the study report implies that outcome was assessed radiographically, it is not stated that this was by independent blind assessment.

h. Follow-up was by record linkage to the Ontario Mortality Database and Cancer Registry so subjects who moved away from Ontario would not be followed up.
