# Supplementary material for: Systematic review with meta-analysis of the epidemiological evidence relating FEV1 decline to lung cancer risk
Source: BMC Cancer. 2012 Oct 27;12:498. doi: 10.1186/1471-2407-12-498 (PMC3573968; doi:10.1186/1471-2407-12-498)
Supplement: Additional file 2 — Others. DOC file summarizes the results for the four studies which satisfied the inclusion/exclusion criteria but were later rejected as estimates of β could not be derived. [file 1471-2407-12-498-S2.doc]

# Additional file 2: Others

As described in the main paper, seven of 33 publications identified as satisfying the inclusion and exclusion criteria were rejected as their results could not be used to derive estimates of β. These describe results from four studies, which are summarized briefly in this document.

## Study in Aalborg, Denmark

## Vestbo *et al*.[41,42] examined a random sample of 876 men aged 46 to 69 years from Aalborg, Denmark in 1974 and followed them until 1985. Both papers related FEV_1_ residuals to risk of respiratory cancer, occurring in 35 of the men, the first [41] reporting a relative risk of 1.15 (95% CI 0.66-1.99) associated with the height-standardised FEV_1_ residual per litre under the expected FEV_1_ given height, after adjustment for age and smoking habits, while the second reported a relative risk of 2.1 (95% CI 1.3-3.4). This study has not been used, partly because of the conflict of results from the two papers, partly as it concerned respiratory cancer rather than lung cancer, and mainly because we were unable to estimate β from the way the results were presented.

## Study of iron miners in France

In 1983 Pham *et al*.[36] reported results of five-year follow-up from 1975 of a group of 1173 iron miners from Lorraine, France. In Table III they noted that mean FEV_1_%P was 87.4 (SD 21.8) in the 13 men dying of lung cancer as compared to 105.1 (SD 16.7) in all the underground workers, a difference reported as significant (p<0.01). β cannot be estimated from these data.

Later, in 1993, Chau *et al*.[47] reported results of 10 years follow-up, during which 33 lung cancers occurred. The abstract reported a relation between a decrease in VC, FEV_1_ and FEV_1_/VC and mortality from lung cancer in heavy smokers or men who had worked underground for more than 20 years. However, the detailed results shown in Table 7 give SMRs (separately for non smokers, smokers of 1-19 pack-years and smokers of 20+ pack-years) comparing men who had:

1. VC, FEV_1_ and FEV_1_/VC higher than predicted value
2. VC lower than predicted value
3. FEV_1_ lower than predicted value
4. FEV_1_/VC lower than predicted value

Though the results showed higher risks in groups (b), (c) and (d) than in group (a) for smokers of 20+ pack-years, they did not allow simple comparison by FEV_1_ and hence estimation of β.

## Study of heavily asbestos-exposed shipyard workers

From a group of 1,500 asbestos workers who had undergone evaluation between 1979 and 1983, Harber *et al*.[29] reported results of a nested case-control study comparing 18 employees who had developed asbestos-associated malignancy with 18 employees individually matched on smoking, age and length of asbestos exposure. 13 of the cases were of lung cancer. The authors only reported mean differences in FEV_1_ residuals and other lung function indices between cases and controls, noting there was a tendency for lung function to be worse in those persons who subsequently developed lung cancer, though this was significant (p<0.05) only for FEV_1_/FVC. β cannot be estimated from the mean difference in FEV_1_ residuals.

## Italian rural cohorts of the Seven Countries Study

Menotti *et al*.[33,34] reported results from the Italian rural cohorts (Crevalcore in Emilia, and Montegiorgio in Marche) of the Seven Countries Study involving 1530 men aged 40 to 59 followed for 25 years from 1960, during which 36 lung cancer cases occurred. Their results were reported in a way that allowed estimation of β as 0.0941 (SE 0.0687), but as they related to forced expiratory volume in ¾ seconds and not 1 second, the estimate has not been included in our main results.
