# Supplementary material for: Systematic review with meta-analysis of the epidemiological evidence relating FEV1 decline to lung cancer risk
Source: BMC Cancer. 2012 Oct 27;12:498. doi: 10.1186/1471-2407-12-498 (PMC3573968; doi:10.1186/1471-2407-12-498)
Supplement: Additional file 3 — Fit. DOC file giving, for each of the blocks considered in Table 4 that include more than two levels, a plot by decline in FEV1%P of the observed RRs (with 95% CIs) and the RRs fitted based on the value of β for that block. The fitted value of β and its SE are shown in the heading for the block. [file 1471-2407-12-498-S3.doc]

Additional file 3: Fit

This file gives, for each of the blocks considered in Table 5 that include more than two levels, a plot comparing the observed lung cancer RRs (with 95% CIs) with those predicted based on the value of β fitted for that block. The fitted value of β and its SE, is shown in the heading for the block. The y-axis is the RR, with the scale running up to 10.00, and upper CIs above 10.00 indicated above open-ended arrows.The x-axis, labelled “Dose vs High FEV_1_%P”, is the reduction in mean FEV_1_%P between that in the comparison group (for which the RR is taken to be 1.0). Exceptionally, in block 5, where the second level had a mean FEV_1_%P slightly higher than that in the comparison group, 10 has been added to the values of “Dose vs High FEV_1_%P”.

FEV1 and Lung Cancer; Plot of Observed and Predicted Relative Risk of Lung Cancer by difference in FEV1%P

Block:Study = 2:CALABR, Beta = 0.0237 (SE = 0.0076)

**Observed RR vs High**

**Predicted RR**

**Dose vs High FEV1%P**

**0.0**

**2.0**

**4.0**

**6.0**

**8.0**

**10.0**

**0**

**4**

**8**

**12**

**16**

**20**

**24**

**28**

**32**

**36**

**40**

**44**

**48**

**52**

**56**

**60**

**64**

**68**

FEV1 and Lung Cancer; Plot of Observed and Predicted Relative Risk of Lung Cancer by difference in FEV1%P

Block:Study = 3:CARET,HI, Beta = 0.0223 (SE = 0.0065)

**Observed RR vs High**

**Predicted RR**

**Dose vs High FEV1%P**

**0.0**

**2.0**

**4.0**

**6.0**

**8.0**

**10.0**

**0**

**4**

**8**

**12**

**16**

**20**

**24**

**28**

**32**

**36**

**40**

**44**

**48**

**52**

**56**

**60**

**64**

**68**

FEV1 and Lung Cancer; Plot of Observed and Predicted Relative Risk of Lung Cancer by difference in FEV1%P

Block:Study = 4:CARET,LO, Beta = 0.0116 (SE = 0.0056)

**Observed RR vs High**

**Predicted RR**

**Dose vs High FEV1%P**

**0.0**

**2.0**

**4.0**

**6.0**

**8.0**

**10.0**

**0**

**4**

**8**

**12**

**16**

**20**

**24**

**28**

**32**

**36**

**40**

**44**

**48**

**52**

**56**

**60**

**64**

**68**

FEV1 and Lung Cancer; Plot of Observed and Predicted Relative Risk of Lung Cancer by difference in FEV1%P

Block:Study = 5:CARTA, Beta = 0.0721 (SE = 0.0490)

**Observed RR vs High**

**Predicted RR**

**Dose vs High FEV1%P + 10**

**0.0**

**2.0**

**4.0**

**6.0**

**8.0**

**10.0**

**0**

**4**

**8**

**12**

**16**

**20**

**24**

**28**

**32**

**36**

**40**

**44**

**48**

**52**

**56**

**60**

**64**

**68**

**15.1**

**19.7**

FEV1 and Lung Cancer; Plot of Observed and Predicted Relative Risk of Lung Cancer by difference in FEV1%P

Block:Study = 6:FINKEL, Beta = 0.0088 (SE = 0.0108)

**Observed RR vs High**

**Predicted RR**

**Dose vs High FEV1%P**

**0.0**

**2.0**

**4.0**

**6.0**

**8.0**

**10.0**

**0**

**4**

**8**

**12**

**16**

**20**

**24**

**28**

**32**

**36**

**40**

**44**

**48**

**52**

**56**

**60**

**64**

**68**

FEV1 and Lung Cancer; Plot of Observed and Predicted Relative Risk of Lung Cancer by difference in FEV1%P

Block:Study = 9:LANGE, Beta = 0.0201 (SE = 0.0042)

**Observed RR vs High**

**Predicted RR**

**Dose vs High FEV1%P**

**0.0**

**2.0**

**4.0**

**6.0**

**8.0**

**10.0**

**0**

**4**

**8**

**12**

**16**

**20**

**24**

**28**

**32**

**36**

**40**

**44**

**48**

**52**

**56**

**60**

**64**

**68**

FEV1 and Lung Cancer; Plot of Observed and Predicted Relative Risk of Lung Cancer by difference in FEV1%P

Block:Study = 12:MRFIT, Beta = 0.0312 (SE = 0.0045)

**Observed RR vs High**

**Predicted RR**

**Dose vs High FEV1%P**

**0.0**

**2.0**

**4.0**

**6.0**

**8.0**

**10.0**

**0**

**4**

**8**

**12**

**16**

**20**

**24**

**28**

**32**

**36**

**40**

**44**

**48**

**52**

**56**

**60**

**64**

**68**

FEV1 and Lung Cancer; Plot of Observed and Predicted Relative Risk of Lung Cancer by difference in FEV1%P

Block:Study = 13:NOMURA, Beta = 0.0178 (SE = 0.0054)

**Observed RR vs High**

**Predicted RR**

**Dose vs High FEV1%P**

**0.0**

**2.0**

**4.0**

**6.0**

**8.0**

**10.0**

**0**

**4**

**8**

**12**

**16**

**20**

**24**

**28**

**32**

**36**

**40**

**44**

**48**

**52**

**56**

**60**

**64**

**68**

FEV1 and Lung Cancer; Plot of Observed and Predicted Relative Risk of Lung Cancer by difference in FEV1%P

Block:Study = 14:PETO, Beta = 0.0184 (SE = 0.0078)

**Observed RR vs High**

**Predicted RR**

**Dose vs High FEV1%P**

**0.0**

**2.0**

**4.0**

**6.0**

**8.0**

**10.0**

**0**

**4**

**8**

**12**

**16**

**20**

**24**

**28**

**32**

**36**

**40**

**44**

**48**

**52**

**56**

**60**

**64**

**68**

FEV1 and Lung Cancer; Plot of Observed and Predicted Relative Risk of Lung Cancer by difference in FEV1%P

Block:Study = 16:RENFRE,M, Beta = 0.0146 (SE = 0.0030)

**Observed RR vs High**

**Predicted RR**

**Dose vs High FEV1%P**

**0.0**

**2.0**

**4.0**

**6.0**

**8.0**

**10.0**

**0**

**4**

**8**

**12**

**16**

**20**

**24**

**28**

**32**

**36**

**40**

**44**

**48**

**52**

**56**

**60**

**64**

**68**

FEV1 and Lung Cancer; Plot of Observed and Predicted Relative Risk of Lung Cancer by difference in FEV1%P

Block:Study = 17:RENFRE,F, Beta = 0.0106 (SE = 0.0052)

**Observed RR vs High**

**Predicted RR**

**Dose vs High FEV1%P**

**0.0**

**2.0**

**4.0**

**6.0**

**8.0**

**10.0**

**0**

**4**

**8**

**12**

**16**

**20**

**24**

**28**

**32**

**36**

**40**

**44**

**48**

**52**

**56**

**60**

**64**

**68**

**10.4**

FEV1 and Lung Cancer; Plot of Observed and Predicted Relative Risk of Lung Cancer by difference in FEV1%P

Block:Study = 19:SPEIZE,M, Beta = 0.0482 (SE = 0.0135)

**Observed RR vs High**

**Predicted RR**

**Dose vs High FEV1%P**

**0.0**

**2.0**

**4.0**

**6.0**

**8.0**

**10.0**

**0**

**4**

**8**

**12**

**16**

**20**

**24**

**28**

**32**

**36**

**40**

**44**

**48**

**52**

**56**

**60**

**64**

**68**

**23.7**

**13.0**

**49.7**

FEV1 and Lung Cancer; Plot of Observed and Predicted Relative Risk of Lung Cancer by difference in FEV1%P

Block:Study = 20:SPEIZE,F, Beta = 0.0540 (SE = 0.0287)

**Observed RR vs High**

**Predicted RR**

**Dose vs High FEV1%P**

**0.0**

**2.0**

**4.0**

**6.0**

**8.0**

**10.0**

**0**

**4**

**8**

**12**

**16**

**20**

**24**

**28**

**32**

**36**

**40**

**44**

**48**

**52**

**56**

**60**

**64**

**68**

**166.3**

**121.2**

**396.8**

FEV1 and Lung Cancer; Plot of Observed and Predicted Relative Risk of Lung Cancer by difference in FEV1%P

Block:Study = 21:STAVEM, Beta = 0.0213 (SE = 0.0081)

**Observed RR vs High**

**Predicted RR**

**Dose vs High FEV1%P**

**0.0**

**2.0**

**4.0**

**6.0**

**8.0**

**10.0**

**0**

**4**

**8**

**12**

**16**

**20**

**24**

**28**

**32**

**36**

**40**

**44**

**48**

**52**

**56**

**60**

**64**

**68**

FEV1 and Lung Cancer; Plot of Observed and Predicted Relative Risk of Lung Cancer by difference in FEV1%P

Block:Study = 24:TOCKMA, Beta = 0.0210 (SE = 0.0104)

**Observed RR vs High**

**Predicted RR**

**Dose vs High FEV1%P**

**0.0**

**2.0**

**4.0**

**6.0**

**8.0**

**10.0**

**0**

**4**

**8**

**12**

**16**

**20**

**24**

**28**

**32**

**36**

**40**

**44**

**48**

**52**

**56**

**60**

**64**

**68**

FEV1 and Lung Cancer; Plot of Observed and Predicted Relative Risk of Lung Cancer by difference in FEV1%P

Block:Study = 25:VANDEN,M,N, Beta = 0.0178 (SE = 0.0130)

**Observed RR vs High**

**Predicted RR**

**Dose vs High FEV1%P**

**0.0**

**2.0**

**4.0**

**6.0**

**8.0**

**10.0**

**0**

**4**

**8**

**12**

**16**

**20**

**24**

**28**

**32**

**36**

**40**

**44**

**48**

**52**

**56**

**60**

**64**

**68**

FEV1 and Lung Cancer; Plot of Observed and Predicted Relative Risk of Lung Cancer by difference in FEV1%P

Block:Study = 26:VANDEN,M,X, Beta = 0.0104 (SE = 0.0067)

**Observed RR vs High**

**Predicted RR**

**Dose vs High FEV1%P**

**0.0**

**2.0**

**4.0**

**6.0**

**8.0**

**10.0**

**0**

**4**

**8**

**12**

**16**

**20**

**24**

**28**

**32**

**36**

**40**

**44**

**48**

**52**

**56**

**60**

**64**

**68**

FEV1 and Lung Cancer; Plot of Observed and Predicted Relative Risk of Lung Cancer by difference in FEV1%P

Block:Study = 27:VANDEN,M,C, Beta = 0.0117 (SE = 0.0033)

**Observed RR vs High**

**Predicted RR**

**Dose vs High FEV1%P**

**0.0**

**2.0**

**4.0**

**6.0**

**8.0**

**10.0**

**0**

**4**

**8**

**12**

**16**

**20**

**24**

**28**

**32**

**36**

**40**

**44**

**48**

**52**

**56**

**60**

**64**

**68**

FEV1 and Lung Cancer; Plot of Observed and Predicted Relative Risk of Lung Cancer by difference in FEV1%P

Block:Study = 28:VANDEN,F,N, Beta = -0.0039 (SE = 0.0159)

**Observed RR vs High**

**Predicted RR**

**Dose vs High FEV1%P**

**0.0**

**2.0**

**4.0**

**6.0**

**8.0**

**10.0**

**0**

**4**

**8**

**12**

**16**

**20**

**24**

**28**

**32**

**36**

**40**

**44**

**48**

**52**

**56**

**60**

**64**

**68**

FEV1 and Lung Cancer; Plot of Observed and Predicted Relative Risk of Lung Cancer by difference in FEV1%P

Block:Study = 29:VANDEN,F,X, Beta = 0.0258 (SE = 0.0112)

**Observed RR vs High**

**Predicted RR**

**Dose vs High FEV1%P**

**0.0**

**2.0**

**4.0**

**6.0**

**8.0**

**10.0**

**0**

**4**

**8**

**12**

**16**

**20**

**24**

**28**

**32**

**36**

**40**

**44**

**48**

**52**

**56**

**60**

**64**

**68**

FEV1 and Lung Cancer; Plot of Observed and Predicted Relative Risk of Lung Cancer by difference in FEV1%P

Block:Study = 30:VANDEN,F,C, Beta = 0.0186 (SE = 0.0041)

**Observed RR vs High**

**Predicted RR**

**Dose vs High FEV1%P**

**0.0**

**2.0**

**4.0**

**6.0**

**8.0**

**10.0**

**0**

**4**

**8**

**12**

**16**

**20**

**24**

**28**

**32**

**36**

**40**

**44**

**48**

**52**

**56**

**60**

**64**

**68**

FEV1 and Lung Cancer; Plot of Observed and Predicted Relative Risk of Lung Cancer by difference in FEV1%P

Block:Study = 31:WILES, Beta = 0.0213 (SE = 0.0076)

**Observed RR vs High**

**Predicted RR**

**Dose vs High FEV1%P**

**0.0**

**2.0**

**4.0**

**6.0**

**8.0**

**10.0**

**0**

**4**

**8**

**12**

**16**

**20**

**24**

**28**

**32**

**36**

**40**

**44**

**48**

**52**

**56**

**60**

**64**

**68**

FEV1 and Lung Cancer; Plot of Observed and Predicted Relative Risk of Lung Cancer by difference in FEV1%P

Block:Study = 32:WILSON, Beta = 0.0081 (SE = 0.0071)

**Observed RR vs High**

**Predicted RR**

**Dose vs High FEV1%P**

**0.0**

**2.0**

**4.0**

**6.0**

**8.0**

**10.0**

**0**

**4**

**8**

**12**

**16**

**20**

**24**

**28**

**32**

**36**

**40**

**44**

**48**

**52**

**56**

**60**

**64**

**68**
